# Collateral benefits of ivermectin mass drug administration designed for malaria against headlice in Mopeia, Mozambique: a cluster randomised controlled trial

**DOI:** 10.1186/s40249-025-01290-z

**Published:** 2025-03-27

**Authors:** Joanna Furnival-Adams, Amelia Houana, Patricia Nicolas, Julia Montaña, Samuel Martinho, Aina Casellas, Hansel Mundaca, Jenisse Mbanze, Arlindo Soares, Saimado Imputiua, Paula Ruiz-Castillo, Marta Ribes, Almudena Sanz, Mussa Mamudo Salé, Antonio Macucha, Eldo Elobolobo, Vegovito Vegove, Victor Mutepa, Humberto Munguambe, Aida Xerinda, Felisbela Materula, Regina Rabinovich, Francisco Saute, Carlos Chaccour

**Affiliations:** 1 IsGlobal, Barcelona Institute for Global Health, Barcelona, Spain; 2 Facultat de Medicina I Ciències de La Salut (Faculty of Medicine and Health Sciences), Universitat de Barcelona (University of Barcelona), Barcelona, Spain; 3 Centro de Investigação em Saúde de Manhiça (Manhica Health Research Centre), Mopeia, Mozambique; 4 Harvard T.H. Chan School of Public Health, Boston, USA; 5 CIBERINFEC, Madrid, Spain; 6 Navarra Center for International Development, Universidad de Navarra, Pamplona, Spain

**Keywords:** Headlice, Ectoparasites, Mass drug administration, Ivermectin, Malaria

## Abstract

**Background:**

Headlice are prevalent worldwide, with a higher burden in rural, lower-middle income settings. They can cause intense itchiness, discomfort, and secondary bacterial infections with potentially serious consequences. Ivermectin is efficacious against headlice, and is also being evaluated as a malaria vector control tool. In this study, we explored risk factors for headlice, and assessed the efficacy of ivermectin mass drug administration (MDA) designed for malaria against headlice.

**Methods:**

We conducted an open-label, assessor-blind, cluster-randomized controlled trial in Mopeia, Mozambique. A single dose of ivermectin was given monthly to eligible humans or humans and livestock (humans: 400 μg/kg, livestock: 1% injectable 200 μg/kg) in 3 consecutive months during the rainy season. The control group received albendazole (humans only). Thirty-nine clusters (13 per arm) were randomly selected for the nested assessment of headlice prevalence. 1341 treated participants were followed up at least once, 1, 2 and 3 months and 382 untreated (ineligible) participants at 3 and 6 months after the first MDA round. Headlice diagnosis was determined by scalp examination. Logistic regression was used to identify risk factors for headlice at baseline, and to estimate the treatment effect at each time point.

**Results:**

A total of 1309 participants were included in the main analysis assessing ivermectin MDA efficacy, and 1332 in the risk factor analysis. The baseline headlice prevalence was 11%. Risk factors included living with a household member with head itch [adjusted odds ratio (a*OR*) = 48.63, 95% confidence interval (*CI*): 28.7–82.3, *P*-value < 0.0001], being female (a*OR* = 2.25, 95% *CI:* 1.33–3.80, *P*-value < 0.01), and using surface water as the main water (a*OR* = 2.37, 95% *CI:* 1.12–5.33, *P*-value = 0.04). The treated population receiving ivermectin had significantly lower odds of having headlice at 3 months compared to those receiving albendazole (a*OR* = 0.19, 95% *CI:* 0.04–0.91, *P*-value = 0.04). There was no indirect effect on headlice among children ineligible for treatment.

**Conclusions:**

In a highly endemic setting, mass drug administration with ivermectin significantly reduces headlice infestation prevalence among those who receive the drug for three sequential months. The lack of effect among untreated, ineligible children implies that additional interventions would be needed to interrupt local transmission.

**Trial registration:**

This study is registered with ClinicalTrials.gov (NCT04966702).

**Graphical Abstract:**

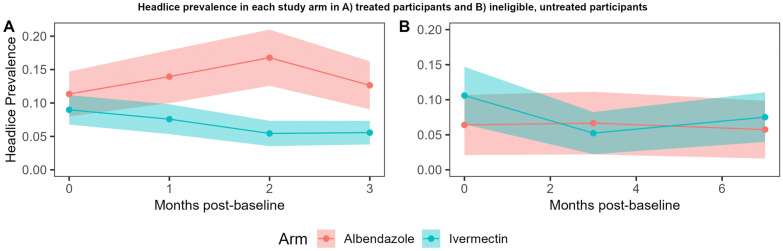

**Supplementary Information:**

The online version contains supplementary material available at 10.1186/s40249-025-01290-z.

## Background

When malaria vector control tools such as indoor residual spraying (IRS) and insecticide treated nets (ITNs) were first being developed, their impact on nuisance parasites such as headlice and bedbugs contributed greatly to their acceptability and uptake [1–3]. Similarly, the early development of resistance in these ectoparasites during dichlorodiphenyltrichloroethane (DDT)-based IRS campaigns led to declines in acceptability and coverage [4, 5]. More recent studies have suggested that resistance in non-target ectoparasites continue to affect acceptability and usage of vector control tools [6–8]. However, the secondary effects on these ectoparasites and off-target diseases are rarely monitored in malaria vector control studies and campaigns.

Headlice infestations are estimated to affect 19% of schoolchildren worldwide, causing stigma and shame among affected individuals, as well as school and work absenteeism [9, 10]. The intense itchiness and discomfort caused by headlice infestations can lead to potentially serious secondary bacterial infections at the lesion sites. Although it has never been incriminated as a vector, some studies have suggested that, like body lice, they may have role in the transmission of *Borrelia* spp., the causal agent of Lyme disease, and relapsing fever [11, 12]. Data on the prevalence and economic burden of headlice is sparse, and the problem of headlice is likely neglected in rural, impoverished areas of the global South, where reporting systems are weak, and other health issues are prioritized [13–15].

A range of topical pediculicidal treatment methods exists including benzyl benzoate, permethrin, lindane, malathion, dimeticone, 1,2-octanediol, and ivermectin [16]. However, many of these are not widely accessible or used to treat headlice in lower-middle income countries. Alternatively, traditional medicines or non-insecticidal treatments can be used. Non-insecticidal treatments include mechanical removal of headlice using a comb, or hands, and head shaving. Resistance to pediculicides has been reported globally, however, most data on this comes from high income countries [17].

Ivermectin mass drug administration (MDA) has been used for decades in the control of endo- and ectoparasites, and it is now under evaluation for use against malaria vectors [18]. If it demonstrates success, it will likely be scaled-up, covering a larger population than currently targeted by the Mectizan Donation Program, which targets onchocerciasis and lymphatic filariasis. Ivermectin is known to be efficacious against headlice when used either topically or orally [19–21]. However, to our knowledge, only one study has assessed the effect of ivermectin MDA (two doses 7 days apart of 200 μg/kg) on headlice [22]. This study reported a significant reduction in headlice prevalence of around 90%. In addition to this, a household-randomized study in Brazil assessed the impact of treating household contacts of headlice cases on their risk of re-infestation, and found that those living in households that received oral ivermectin were significantly more likely to be headlice free after 60 days [23]. Finally, a pilot study in Australia assessed the effect of headlice screening and treating cases and siblings within a school with oral ivermectin versus topical treatment, and the findings suggest that oral ivermectin is more effective than topical treatment [24]. The results from these studies suggest that similar effects may be observed in the context of ivermectin MDA for malaria in areas where headlice are prevalent.

Within a cluster-randomised trial that took place in Mopeia, Mozambique, assessing ivermectin MDA for malaria control, we assessed its impact on headlice prevalence, at both individual and community level, as well as the risk factors for having headlice, and compared the effects of the different treatment arms on headlice.

## Methods

### Study setting and design

The study took place between March and October 2022, in Mopeia, a rural district in Zambezia Province, central Mozambique, which remains highly endemic for malaria despite dual coverage with IRS and ITNs [25]. It has a population of approximately 131,000, and covers an area of 7671 km^2^ [26].

The study was nested within a three-arm cluster-randomised controlled trial, aiming to assess the effect of ivermectin MDA on malaria transmission [27]. Each cluster consisted of a core area in which at least 35 children under 5 lived, and a buffer area with a radius of 400 m around the core. Written consent was obtained from all participants or their legal guardian. The following interventions were given once per month for three consecutive months: (a) ivermectin in humans: a single dose of 400 μg/kg in eligible humans; (b) ivermectin in humans and livestock: a single dose of 400 μg/kg in eligible humans plus 1% injectable ivermectin at a dose of 200 μg/kg to all pigs/cattle in the cluster; (c) control: albendazole was given to humans only at a single dose of 400 mg.

Ivermectin was purchased from Merck Sharp & Dohme (Haarlem, the Netherlands) as Stromectrol® and supplied in 3 mg tablets. Albendazole was purchased from Cipla (Mumbai, India) as 400 mg chewable tablets. All study drugs were delivered door-to-door and drug administration was directly observed by the responsible fieldworker. Within the main Broad One Health Endectocide-based Malaria Intervention in Africa (BOHEMIA) trial, two cohorts were followed up; the safety cohort, and the efficacy cohort. The safety cohort consisted of individuals who met the following criteria: weighing over 15 kg, ability to consent, and a negative pregnancy test for women aged between 13 and 49; and exclusion: known hypersensitivity to ivermectin or albendazole, risk of *Loa loa* as assessed by travel history, pregnancy, lactating in the first week postpartum, currently participating in another clinical trial, unwilling to consent or adhere to study procedures, severely ill, currently under treatment with inhibitors of cytochrome P450 3A or P-glycoprotein (see Supplementary File 1 for the full list of excluded drugs). The purpose of the safety cohort was to serve as delivery vehicle for the insecticidal intervention and to monitor the safety of the treatment regime. These participants were followed up at 1, 2 and 3 months after the first round of MDA.

The efficacy cohort consisted of children under 5 (the primary age group affected by malaria in the region). The purpose of this cohort was to assess the impact of the intervention on malaria. Participants in this cohort were followed up at 3 and 6 months after the first round of MDA.

This headlice sub-study included randomly selected participants from both cohorts. In the analysis, the safety cohort and any participants from the efficacy cohort who had taken ivermectin at least once were categorised as “treated”, and participants from the efficacy cohort who had not taken ivermectin were categorised as “untreated” regardless of their assignment arm.

### Randomisation and sampling

During October 2020 to November 2021, a local demography survey took place under a separate protocol, where all households were mapped and the population was enumerated [26]. Using this data, clusters were created, and randomized 1:1:1 to each intervention using a computer-generated random sequence. Study investigators were masked from the treatment allocation during the study, however, the study participants were aware of which intervention they received. Following treatment allocation, 13 clusters per arm (39 clusters in total) were randomly selected for follow-up of headlice. Within these clusters, all children enrolled into the efficacy cohort of the main study, and 40 participants enrolled into the safety cohort of the main study were randomly selected for inclusion in this study. Participants from the efficacy cohort were followed up at 3 and 6 months after the first round of MDA. Participants from the safety cohort were followed up 1, 2 and 3 months after the first round of MDA.

### Outcomes

The primary outcome was headlice prevalence at 3 months. Secondary outcomes were headlice prevalence at 3 months in treated participants, and at 3 and 6 months in untreated participants.

As part of the main malaria trial, adverse events, and severe adverse events were monitored to assess the safety of the intervention. A Data Safety Monitoring Board was constituted, approved the protocol, received regular reports and met periodically throughout the study period. The safety data will be reported in a separate manuscript.

Fieldworkers were trained in headlice detection over a 2-day period by Joanna Furnival-Adams and Amelia Houana through power point presentations, exercises using a tablet, and written assessments. In the field, participant scalps were inspected visually in daylight or under a torchlight, and signs of headlice (live lice or eggs) were recorded. In the main analysis, only participants with live headlice were categorized as having headlice. Those found infested were offered a referral to the health facility.

### Risk factor analysis variables

Baseline data on headlice, and data from a demographic survey that took place prior to the start of the trial were used to conduct the risk factor analysis. Household wealth index was calculated using a formula detailed in Xie et al. [28]. All variables were determined through a pre-designed questionnaire. Bed net use was a binary variable, assessed by asking the participant or their parent/guardian whether they slept under a bed net the previous night. Details of how water sources were categorised are provided in Supplementary File 2 [29]. Open defecation refers to those whose household does not have a sanitation facility available to them.

### Statistical analysis

The sample size of this nested study was calculated to detect a significant difference between the control and the treatment arms in scabies prevalence, which was another outcome that will be reported in a separate publication (Furnival-Adams et al., *submitted*). Assuming a 7% baseline scabies prevalence in the control group, a cluster size of 40 participants with 13 clusters per arm was required to detect a 60% effect size with 80% power at 5% significance and a kappa of 0.25.

For the risk factor analysis, logistic regression using the lme4 R package (v1.1–26) was used to conduct bivariate and multivariate analyses in the R programming language, version 4.2.2 (R Foundation for Statistical Computing, Vienna, Austria). In all analyses, odds ratios or adjusted odds ratios (a*OR*s) with 95% confidence interval (*CI)* were calculated. All variables associated with headlice in bivariate analysis (*P* < 0.10) were assessed for inclusion in multivariate analysis. Variables that were statistically significantly associated with headlice (*P* < 0.05) were included in the multivariate model.

To assess the treatment effect, we compared headlice prevalence in the control versus the treatment arms at each time point, using logistic regression model. To account for correlation between clusters, we used generalised estimating equations to fit each logistic model of prevalence to the available data. Known risk factors of headlice, age, sex and wealth, were included as covariates in the adjusted models. These data were collected during the census survey that took place prior to the start of the trial. Intervention effects were expressed as adjusted odds ratios. After analysing the data, we found no statistically significant difference between the two treatment arms (human and human + livestock) existed, and therefore pooled the two intervention arms. Statistical significance was considered when the *P*-value was < 0.05 using to the Wald test.

This study was conducted in accordance with the CONSORT guidelines for reporting randomized controlled trials (Supplementary File 3) [30].

## Results

### Enrolment and baseline characteristics of the study population

Between March 2022 and October 2022, 102 clusters were enrolled into the main malaria trial, and of these, 39 clusters were randomly selected for this nested headlice study. 1500 participants were randomly selected to be included in this nested study from the participants enrolled into the main trial, and 1309 were included in the final analysis. The estimated treatment coverage ranged from 50% to 67% per round in eligible participants, and 43% to 52% in the overall population (eligible and ineligible to take ivermectin) (Supplementary File 4). The coverage was similar across arms and across all visits. Further information on the cluster and participant selection process is detailed in a separate publication [31].

At baseline, characteristics of the households and participants were similar across all three intervention arms (Table 1). The median age of participants who took the study drug was 19 years old, and it was 3 years old in the ineligible population. The sex distribution was balanced across all arms in both cohorts. The majority of participants were categorised as being in Rank 2 or 3 according to the wealth rank index in both cohorts. The overall baseline prevalence of headlice was 9.8% in the treated cohort and 9.1% in the untreated cohort.

**Table 1:** Baseline sociodemographic characteristics of the study population in the treated and untreated population in each study arm

Variable	Humans	Humans and livestock	Control
Treated			
Number of clusters	13	13	13
Number of participants	307	347	348
Household wealth index rank			
Rank 1^a^	56/307 (18.2%)	27/344 (7.8%)	42/346 (12.1%)
Rank 2	62/307 (20.2%)	101/344 (29.4%)	97/346 (28.0%)
Rank 3	78/307 (50.6%)	79/344 (23.0%)	81/346 (23.4%)
Rank 4	46/307 (15.0%)	65/344 (18.6%)	82/346 (23.7%)
Rank 5	65/307 (21.2%)	72/344 (20.9%)	44/346 (12.7%)
Median age in years (IQR) [*n*]	20.81 (9.31–39.40) [307]	18.90 (7.92–34.47) [347]	18.90 (7.92–34.47) [347]
Sex: female	155/307 (50.5%)	167/347 (48.1%)	166/348 (47.7%)
Median weight (*SD*) [*n*]	40 (25) [307]	40 (30) [347]	40 (28) [348]
Headlice prevalence	23/304 (7.6%)	35/347 (10.1%)	39/344 (11.3%)
Untreated, ineligible under 5			
Number of clusters	13	13	13
Number of participants	111	106	125
Household wealth index rank			
Rank 1^a^	13/108 (12.0%)	5/104 (4.8%)	13/123 (10.6%)
Rank 2	23/108 (21.3%)	23/104 (22.1%)	27/123 (22.0%)
Rank 3	19/108 (17.6%)	27/104 (26.0%)	40/123 (32.5%)
Rank 4	17/108 (15.7%)	25/104 (24.0)	28/123 (22.8%)
Rank 5	36/108 (33.3)	24/104 (23.1)	15/123 (12.2%)
Median age in years (IQR) [*n*]	2.8 (1.7–3.9) [111]	2.8 (1.8–4.0) [106]	2.8 (2.0–3.8) [125]
Sex: female	61/111 (55.0%)	52/106 (49.0%)	59/125 (47.2%)
Median weight (kg) (*SD*) [*n*]	11 (3) [111]	11 (3) [106]	11 (4) [125]
Headlice prevalence	13/111 (11.7%)	10/106 (9.4%)	8/125 (6.4%)

^a^ Wealthiest

*SD* standard deviation, *IQR* interquartile range

### Headlice risk factors

The risk factors identified for headlice infestation at baseline are shown in Table 2.

**Table 2:** Sociodemographic and water and sanitation-related risk factors for headlice infestation (bivariate analysis) at baseline

Variable	*n/N*	Prevalence	*OR* (95% *CI*)	*P*-value
Sex				
Male	47/678	6.93 (5.14–9.11)	REF	
Female	81/654	12.38 (9.96–15.16)	1.90 (1.3–2.8)	**0.0009**
Age, years				
0–4	26/310	6.93 (5.14–9.11)	REF	
4–11	39/355	12.38 (9.96–15.16)	1.90 (1.3–2.8)	0.261
11–29	34/343	6.93 (5.14–9.11)	REF	0.500
> 29	29/324	12.38 (9.96–15.16)	1.90 (1.3–2.8)	0.801
Another member of the household has an itchy head				
Yes	101/181	55.8 (48.2–63.2)	52.6 (32.5–85.0)	** < 0.0001**
No	27/1,151	2.3 (1.6–3.4)	REF	
Household size (members)				
< 5	76/699	10.87 (8.66–13.42)	REF	
> 5	52/633	8.21 (6.20–10.63)	0.73 (0.51–1.06)	0.10
Wealth index rank				
Rank 1^c^	17/154	11.04 (6.56–17.09)	REF	
Rank 2	27/335	8.06 (5.38–11.51)	0.75 (0.38–1.51)	0.42
Rank 3	26/325	8.00 (5.29–11.50)	0.69 (0.34–1.40)	0.31
Rank 4	29/261	11.11 (7.57–15.57)	1.15 (0.58–2.28)	0.69
Rank 5	29/252	11.51 (7.84–16.11)	1.04 (0.52–2.08)	0.91
Animal ownership^a^				
Yes	42/454	9.25 (6.75–12.30)	0.94 (0.63–1.39)	0.76
No	79/808	9.78 (7.82–12.04)	REF	
Open defecation^b^				
Yes	81/675	12.00 (9.64–14.69)	1.86 (1.26–2.78)	0.002
No	40/588	6.80 (4.90–9.15)	REF	
Water source				
Safely managed	12/97	12.37 (6.56–20.61)	1.99 (0.99–3.98)	0.053
Basic	35/528	6.63 (4.66–9.10)	REF	
Limited	18/194	9.28 (5.59–14.27)	1.44 (0.80–2.61)	0.228
Unimproved	25/233	10.73 (7.07–15.43)	1.69 (0.99–2.90)	0.055
Surface	20/99	20.20 (12.80–29.46)	3.57 (1.96–6.49)	< 0.0001
Bed net use				
Yes	89/871	10.22 (8.29–12.42)	1.45 (0.90–2.35)	0.124
No	23/317	7.26 (4.65–10.69)	REF	

^a^Any of the following animals: cats, dogs, chickens, cattle, pigs, goats or sheep ^b^we planned to classify sanitation according to the DHS/WHO as Safely Managed, Basic, Limited, Unimproved, or Open Defecation, however, only 12 participants were classified in the former 3 categories. We therefore chose to classify participants according to whether they practised open defecation. ^c^Wealthiest, *OR* odds ratio, *CI* confidence interval, *REF* reference

Of the covariates assessed, four were significantly associated with headlice. Having another household member with an itchy head, female sex, practising open defecation, and using surface water as the principal water source were each associated with having headlice.

In the multivariate regression analysis, having another household member with an itchy head (a*OR* = 48.63, 95% *CI:* 28.7–82.3, *P*-value < 0.0001), being female (a*OR* = 2.25, 95% *CI:* 1.33–3.80, *P*-value < 0.01) and using surface water as the main water source (a*OR* = 2.37, 95% *CI:* 1.12–5.33, *P*-value = 0.04) were the strongest predictors of headlice. Practising open defecation was considered for inclusion in the model, however, it was no longer statistically significant in the multivariate model.

### Treatment seeking behaviour in participants with headlice

Regarding treatment-seeking, 13.3% (95% *CI:* 11.5–15.2, *n* = 1345) of participants reported having headlice in the previous month. Of these participants, 3.9% (95% *CI:* 1.6–7.9, *n* = 179) sought treatment, and 71.4% (95% *CI:* 29.0–96.3, *n* = 7) of those who sought treatment reported it to be effective.

### The efficacy of ivermectin MDA against headlice

There were lower odds of having headlice in the overall populations of the ivermectin arms compared to the control arm 3 months after the first dosing (a*OR* = 0.27, 95% *CI:* 0.06–1.15) (Table 3). Whilst there was a strong tendency towards a reduction in headlice in the ivermectin arms, this difference was not statistically significant.

**Table 3:** Effect of ivermectin MDA on headlice prevalence at 3 months in the overall population (both treated and untreated)

Population	*n/N*	Headlice prevalence, % (95% *CI*)	Unadjusted *OR* (95% *CI*)	*P*-value	Adjusted OR* (95% *CI*)	*P*-value
Control						
3 months	50/452	11.06 (8.32–14.32)	–	–	–	–
Ivermectin arms pooled						
3 months	47/857	5.48 (4.06–7.22)	0.59 (0.16–2.16)	0.43	0.27 (0.06–1.15)	0.08

^*^Adjusted for baseline headlice prevalence, age, sex and wealth. – means not applicable, *OR* odds ratio, *MDA* mass drug administration

**Fig. 1:**
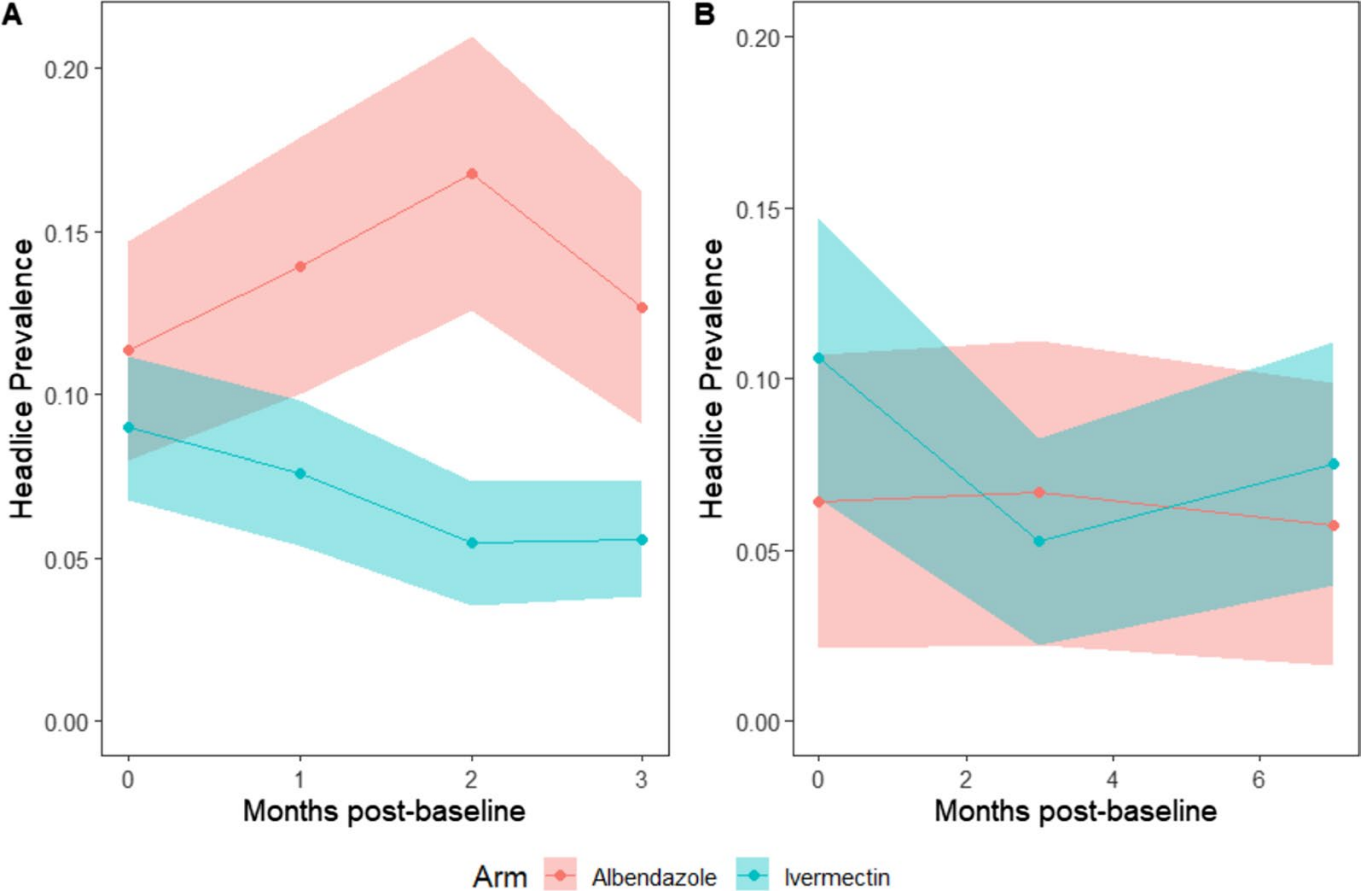
Headlice prevalence in albendazole and ivermectin arms in **A**) treated participants, and **B**) ineligible, untreated participants

In participants who took ivermectin, there was a significant difference in the odds of having headlice prevalence between arms, with significantly lower odds of having headlice in the intervention arms after 3 months in participants who took the drug (a*OR* = 0.19, 95% *CI:* 0.04–0.91) (Table 4) (Fig. 1). The effect on headlice was statistically significant after 2 months, and the effect size gradually increased after each consecutive month. There was no significant difference when comparing the two ivermectin arms, therefore we pooled the data from these arms. We found no evidence of an indirect impact of ivermectin MDA on headlice in the ineligible, untreated population who did not take the study drug at any time point (Supplementary File 5).

**Table 4:** Direct protection in treated participants

Population	*n/N*	Headlice prevalence, % (95% *CI*)	Unadjusted *OR* (95% *CI*)	*P*-value	Adjusted *OR*^a^ (95% *CI*)	*P*-value
Control						
1 month	41/294	13.95 (10.20–18.44)	–	–	1 (REF)	–
2 months	51/304	16.78 (12.75–21.46)	–	–	1 (REF)	–
3 months	42/332	12.65 (9.27–16.71)	–	–	1 (REF)	–
Ivermectin arms pooled						
1 month	41/541	7.58 (5.49–10.14)	0.59 (0.21–1.62)	0.303	0.41 (0.13–1.25)	0.116
2 months	30/551	5.44 (3.70–7.68)	0.37 (0.12–1.11)	0.077	0.29 (0.10–0.81)	0.018
3 months	36/647	5.56 (3.93–7.62)	0.51 (0.15–1.74)	0.285	0.19 (0.04–0.91)	0.038

^a^Adjusted for baseline headlice prevalence, age, sex and wealth. – means not applicable. *OR* odds ratio

## Discussion

In this study, we described headlice epidemiology in Mopeia, Mozambique, and assessed the effect of ivermectin MDA on headlice prevalence within a cluster-randomised controlled trial. We found that the baseline prevalence of headlice was approximately 10% in the study area, and having headlice was significantly associated with having another household member with an itchy head; being female; and having surface water as the main water source. We also found that only a small proportion of those who believed they have headlice sought treatment. Finally, ivermectin MDA designed for malaria (with a higher dosage and frequency of dosages compared to those used for MDA programmes for onchocerciasis and lymphatic filariasis), significantly reduced the odds of having headlice by around 78% after 3 months in the population who took the study drug. However, in participants who were ineligible to take ivermectin, no effect was observed. Within the main trial, there was no statistically significant difference in the rate of severe adverse events between the ivermectin and albendazole arms.

The findings show that headlice are common in the district of Mopeia, and that infestations are clustered within households. Regions with inadequate access to safe water and sanitation facilities, such as Mopeia, may be at higher risk of headlice. Whilst headlice are primarily spread through person-to-person contact, access to clean water, sanitation and hygiene, and regular washing likely reduces transmission through mechanical removal of headlice [32]. Poor water and sanitation conditions may therefore indirectly propagate the spread of headlice. These risk factors have also been identified in previous studies [9, 32, 33]. Being female is a risk factor for headlice in many settings, most likely due to the tendency for females to have longer hair [34, 35]. The low levels of treatment-seeking behaviour may suggest that treatments are not available or affordable, or that headlice are not perceived as a health issue amongst the community. Further research would be needed to understand this better.

The significant impact that we observed on headlice for those who took ivermectin may have an important role in improving the quality of life of participants, as well as the acceptability of the malaria intervention, and its perceived efficacy. The effect we observed in the treated cohort is consistent with a previous before–after study that took place in the Solomon Islands that indicated a relative reduction of 70% [22]. However, the lack of an effect in those not taking ivermectin reduces its potential, as a stand-alone intervention, to control headlice at a community level. Ivermectin is not ovicidal, therefore a second dose of ivermectin is typically recommended 7 days after the first dose to target eggs that are not targeted with the first dose. However, in this study, only a single dose was given per month, which suggests that a single dose can effectively treat headlice. Further research on the pharmacokinetics of 400 μg/kg ivermectin for headlice would be needed to understand this effect.

Our study has some limitations. The fieldworkers examining participants’ scalps were not able to use a comb, so it is possible that we underestimated the prevalence of headlice. The follow-up period of 3 months is relatively short; it is not clear whether the reduction in headlice would be sustained beyond the follow-up points in our study.

## Conclusions

In this study, we identified some risk factors of headlice, namely having another household member with an itchy head; being female; and having surface water as the main water source. We also found that ivermectin MDA designed for malaria offers an opportunity to target headlice infestations in the eligible population. This implies that there are secondary benefits to implementing ivermectin MDA for malaria in co-endemic areas, which has implications for the quality of life amongst the community, as well as the acceptability of the intervention.

## Supplementary Information

Supplementary Material 1

Supplementary Material 2

Supplementary Material 3

Supplementary Material 4

Supplementary Material 5

Supplementary Material 6

Supplementary Material 7

## Data Availability

Anonymized individual data relevant to this article is available in Supplementary File 6, and the corresponding code is in Supplementary File 7.

## Abbreviations

MDA: Mass drug administration
IRS: Indoor residual spraying
ITN: Insecticide-treated net
DDT: Dichlorodiphenyltrichloroethane
CYP3A: Cytochrome P450A
*CI*: Confidence interval
*OR*: Odds ratio
P-gp: P-glycoprotein
GEE: Generalized estimating equation

## References

[1] Lindsay SW, Snow RW, Armstrong JRM, Greenwood BM. Permethrin-impregnated bednets reduce nuisance arthropods in Gambian houses. Med Vet Entomol. 1989;3:377–83. 2519687 10.1111/j.1365-2915.1989.tb00244.x

[2] Temu EA, Minjas JN, Shiff CJ, Majala A. Bedbug control by permethrin-impregnated bednets in Tanzania. Med Vet Entomol. 1999;13:457–9. 10608237 10.1046/j.1365-2915.1999.00194.x

[3] Sharma SK, Upadhyay AK, Haque MA, Padhan K, Tyagi PK, Ansari MA, et al. Wash resistance and bioefficacy of Olyset net—a long-lasting insecticide-treated mosquito net against malaria vectors and nontarget household pests. J Med Entomol. 2006;43(5):884–8. 17017224 10.1603/0022-2585(2006)43[884:wraboo]2.0.co;2

[4] Gunasekaran K, Sahu SS, Jambulingam P, Das PK. DDT indoor residual spray, still an effective tool to control *Anopheles fluviatilis*-transmitted *Plasmodium falciparum* malaria in India. Trop Med Int Heal. 2005;10(2):160–8. 10.1111/j.1365-3156.2004.01369.x 15679559

[5] Myamba J, Maxwell CA, Asidi A, Curtis CF. Pyrethroid resistance in tropical bedbugs, *Cimex hemipterus*, associated with use of treated bednets. Med Vet Entomol. 2002;16:448–51. 12510899 10.1046/j.1365-2915.2002.00389.x

[6] Malede A, Aemero M, Gari SR, Kloos H, Alemu K. Barriers of persistent long-lasting insecticidal nets utilization in villages around Lake Tana, Northwest Ethiopia: a qualitative study. BMC Public Health. 2019;19(1):1–11. 31619208 10.1186/s12889-019-7692-2 PMC6796332

[7] Liheluka EA, Massawe IS, Chiduo MG, Mandara CI, Chacky F, Ndekuka L, et al. Community knowledge, attitude, practices and beliefs associated with persistence of malaria transmission in North-western and Southern regions of Tanzania. Malar J. 2023;22(1):1–16. 37817185 10.1186/s12936-023-04738-5 PMC10563328

[8] Parker W, Pennas T, Kommwa I. Community health priorities: lessons for malaria prevention from Balaka district. Malawi Malawi Med J. 2018;30(2):99–102. 30627337 10.4314/mmj.v30i2.9 PMC6307075

[9] Jamani S, Rodríguez C, Rueda MM, Matamoros G, Canales M, Bearman G, et al. Head lice infestations in rural Honduras: the need for an integrated approach to control neglected tropical diseases. Int J Dermatol. 2019;58(5):548–56. 30549003 10.1111/ijd.14331

[10] Hatam-Nahavandi K, Ahmadpour E, Pashazadeh F, Dezhkam A, Zarean M, Rafiei-Sefiddashti R, et al. Pediculosis capitis among school-age students worldwide as an emerging public health concern: a systematic review and meta-analysis of past five decades. Parasitol Res. 2020;119(10):3125–43. 32803332 10.1007/s00436-020-06847-5

[11] Amanzougaghene N, Akiana J, Mongo Ndombe G, Davoust B, Nsana NS, Parra HJ, et al. Head lice of pygmies reveal the presence of relapsing fever borreliae in the Republic of Congo. PLoS Negl Trop Dis. 2016;10(12): e0005142. 27911894 10.1371/journal.pntd.0005142 PMC5135033

[12] Feldmeier H. Head lice as vectors of pathogenic microorganisms. Trop Med Health. 2023;51(1):53. 37730694 10.1186/s41182-023-00545-5 PMC10510260

[13] Coscione S, Kositz C, Marks M. Head lice: an under-recognized tropical problem. Am J Trop Med Hyg. 2017;97(6):1636–7. 29187278 10.4269/ajtmh.17-0656 PMC5805079

[14] Dagne H, Biya AA, Tirfie A, Yallew WW, Dagnew B. Prevalence of pediculosis capitis and associated factors among schoolchildren in Woreta town, northwest Ethiopia. BMC Res Notes. 2019;12(1):1–6. 31362792 10.1186/s13104-019-4521-8 PMC6668114

[15] Manrique-Saide P, Pavía-Ruz N, Rodríguez-Buenfil JC, Herrera Herrera R, Gómez-Ruiz P, Pilger D. Prevalence of pediculosis capitis in children from a rural school in Yucatan, Mexico. Rev Inst Med Trop Sao Paulo. 2011;53(6):325–7. 22183456 10.1590/s0036-46652011000600005

[16] Sangaré AK, Doumbo OK, Raoult D. Management and treatment of human lice. Biomed Res Int. 2016;2016:8962685. 27529073 10.1155/2016/8962685 PMC4978820

[17] Durand R, Bouvresse S, Berdjane Z, Izri A, Chosidow O, Clark JM. Insecticide resistance in head lice: clinical, parasitological and genetic aspects. Clin Microbiol Infect. 2012;18(4):338–44. 22429458 10.1111/j.1469-0691.2012.03806.x

[18] Billingsley P, Binka F, Chaccour C, Foy BD, Gold S, Gonzalez-Silva M, et al. A roadmap for the development of ivermectin as a complementary malaria vector control tool. Am J Trop Med Hyg. 2020;102(2_suppl):3–24. 31971144 10.4269/ajtmh.19-0620 PMC7008306

[19] Deeks LS, Naunton M, Currie MJ, Bowden FJ. Topical ivermectin 0.5% lotion for treatment of head lice. Ann Pharmacother. 2013;47(9):1161–7. 24259731 10.1177/1060028013500645

[20] Chosidow O, Giraudeau B, Cottrell J, Izri A, Hofmann R, Mann SG, et al. Oral ivermectin versus malathion lotion for difficult-to-treat head lice. N Engl J Med. 2010;362(10):896–905. 20220184 10.1056/NEJMoa0905471

[21] Nofal A. Oral ivermectin for head lice: a comparison with 0.5 % topical malathion lotion. J Dtsch Dermatol Ges. 2010;8(12):985–8. 20718901 10.1111/j.1610-0387.2010.07487.x

[22] Coscione S, Esau T, Kekeubata E, Diau J, Asugeni R, MacLaren D, et al. Impact of ivermectin administered for scabies treatment on the prevalence of head lice in Atoifi, Solomon Islands. PLoS Negl Trop Dis. 2018;12(9): e0006825. 30252856 10.1371/journal.pntd.0006825 PMC6173439

[23] Pilger D, Heukelbach J, Khakban A, Oliveira FA, Fengler G, Feldmeier H. Household-wide ivermectin treatment for head lice in an impoverished community: randomized observer-blinded controlled trial. Bull World Health Organ. 2010;88(2):90–6. 20428365 10.2471/BLT.08.051656 PMC2814473

[24] Currie MJ, Reynolds GJ, Glasgow NJ, Bowden FJ. A pilot study of the use of oral ivermectin to treat head lice in primary school students in Australia. Pediatr Dermatol. 2010;27(6):595–9. 21138467 10.1111/j.1525-1470.2010.01317.x

[25] Chaccour C, Zulliger R, Wagman J, Casellas A, Nacima A, Elobolobo E, et al. Incremental impact on malaria incidence following indoor residual spraying in a highly endemic area with high standard ITN access in Mozambique: results from a cluster-randomized study. Malar J. 2021;20(1):1–15. 33568137 10.1186/s12936-021-03611-7 PMC7877039

[26] Ruiz-Castillo P, Imputiua S, Xie K, Elobolobo E, Nicolas P, Montaña J, et al. BOHEMIA a cluster randomized trial to assess the impact of an endectocide-based one health approach to malaria in Mozambique: baseline demographics and key malaria indicators. Malar J. 2023;22(1):1–12. 37271818 10.1186/s12936-023-04605-3 PMC10239551

[27] Chaccour C, Casellas A, Hammann F, Ruiz-Castillo P, Nicolas P, Montaña J, et al. BOHEMIA: Broad One Health Endectocide-based Malaria Intervention in Africa—a phase III cluster-randomized, open-label, clinical trial to study the safety and efficacy of ivermectin mass drug administration to reduce malaria transmission in two African set. Trials. 2023;24(1):1–16. 36810194 10.1186/s13063-023-07098-2 PMC9942013

[28] Xie K, Marathe A, Deng X, Ruiz-Castillo P, Imputiua S, Elobolobo E, et al. Alternative approaches for creating a wealth index: the case of Mozambique. BMJ Glob Heal. 2023;8:12639. 10.1136/bmjgh-2023-012639 PMC10465889 37643807

[29] UNICEF and WHO. Progress on drinking water and sanitation. UNICEF and WHO; 2012. p. 1–58.

[30] Moher D, Hopewell S, Schulz KF, Montori V, Gøtzsche PC, Devereaux PJ, et al. CONSORT 2010 explanation and elaboration: updated guidelines for reporting parallel group randomised trials. BMJ. 2010;340:869. 10.1136/bmj.c869 PMC2844943 20332511

[31] Furnival-Adams J, Houana A, Saute F, Rudd M, Nicolas P, Montaña J, et al. Direct and indirect protection against scabies through ivermectin mass drug administration designed for malaria in Mozambique: a cluster randomised controlled trial; 2024. 10.2139/ssrn.4952002 10.1016/j.lanmic.2025.101189 41274299

[32] Mahmud S, Pappas G, Hadden WC. Prevalence of head lice and hygiene practices among women over twelve years of age in Sindh, Balochistan, and North West Frontier Province: National Health Survey of Pakistan, 1990–1994. Parasit Vectors. 2011;4(1):1–10. 21288357 10.1186/1756-3305-4-11 PMC3040706

[33] Munusamy H, Elsa E, Murhandarwati H, Rahmah S. The relationship between the prevalence of head lice infestation with hygiene and knowledge among the rural school children in Yogyakarta. Trop Med J. 2014;01(02):102–9.

[34] Suleman M, Jabeen N. Head lice infestation in some urban localities of NWFP, Pakistan. Ann Trop Med Parasitol. 1989;83(5):539–47. 2619367 10.1080/00034983.1989.11812384

[35] Counahan M, Andrews R, Büttner P, Byrnes G, Speare R. Head lice prevalence in primary schools in Victoria, Australia. J Paedriatr Child Heal. 2004;40:616–9. 10.1111/j.1440-1754.2004.00486.x 15469530

