# Supplementary material for: Collateral benefits of ivermectin mass drug administration designed for malaria against headlice in Mopeia, Mozambique: a cluster randomised controlled trial
Source: Infect Dis Poverty. 2025 Mar 27;14:25. doi: 10.1186/s40249-025-01290-z (PMC11948683; doi:10.1186/s40249-025-01290-z)
Supplement: Supplementary file 1 — Supplementary Material 1 [file 40249_2025_1290_MOESM1_ESM.docx]

Supplementary file 1. List of concomitant drugs

| Class of drug | Drug name |
| --- | --- |
| Anti-HIV therapy | ALUVIA (Lopinavir/Ritonavir) |
|  | Norvir (Ritonavir) |
|  | Kaletra (Lopinavir/Ritonavir) |
|  | ALLTERA (Lopinavir/Ritonavir) |
|  | Indinavir |
| Anti-arrhythmic/hypertensive medicine | Spironolactone |
|  | Quinidine |
|  | Amiodarone |
|  | Diltiazem |
|  | Verapamil |
| Antibiotic macrolides | Erythromycin |
|  | Clarithromycin |
| Anticoagulants | Warfarin |
| Antifungal agents | Itraconazole |
|  | Ketoconazole |
| H-2 blockers | Cimetidine |
| Immunosuppressants | Tacrolimus |
|  | Cyclosporine |
| Steroids | Dexamethasone |
