# Supplementary material for: Collateral benefits of ivermectin mass drug administration designed for malaria against headlice in Mopeia, Mozambique: a cluster randomised controlled trial
Source: Infect Dis Poverty. 2025 Mar 27;14:25. doi: 10.1186/s40249-025-01290-z (PMC11948683; doi:10.1186/s40249-025-01290-z)
Supplement: Supplementary file 2 — Supplementary Material 2 [file 40249_2025_1290_MOESM2_ESM.docx]

Additional File 2. Classification of water source according to WHO/UNICEF

|  | Definition (According to the WHO/UNICEF (33)) |
| --- | --- |
| Safely Managed | Drinking water from an improved water source that is located on premises, available when needed and free from faecal and priority chemical contamination |
| Basic | Drinking water from an improved source, provided collection time is not more than 30 minutes for a round trip, including queuing |
| Limited | Drinking water from an improved source for which collection  time exceeds 30 minutes for a round trip, including queuing |
| Unimproved | Drinking water from an unprotected dug well or unprotected spring |
| Surface | Drinking water directly from a river, dam, lake, pond, stream, canal or irrigation canal |
