# Supplementary material for: Collateral benefits of ivermectin mass drug administration designed for malaria against headlice in Mopeia, Mozambique: a cluster randomised controlled trial
Source: Infect Dis Poverty. 2025 Mar 27;14:25. doi: 10.1186/s40249-025-01290-z (PMC11948683; doi:10.1186/s40249-025-01290-z)
Supplement: Supplementary file 4 — Supplementary Material 4 [file 40249_2025_1290_MOESM4_ESM.docx]

Additional File 4. Coverage in the eligible and ineligible population

|  | Ivermectin arms | | | Albendazole | | |
| --- | --- | --- | --- | --- | --- | --- |
| MDA round | 1 | 2 | 3 | 1 | 2 | 3 |
| Eligible population | 4867/8844 (56.94%) | 4980/7975 (62.45%) | 5204/7718 (67.43%) | 2853/5666 (50.35%) | 3227/5115 (63.09%) | 3299/4954 (66.59%) |
| Total population (eligible and ineligible) | 4867/10313 (47.19%) | 4980/10120 (49.21%) | 5204/10111 (51.47%) | 2853/6625 (43.06%) | 3227/6386 (50.53%) | 3299/6398 (51.56%) |
