# Supplementary material for: Collateral benefits of ivermectin mass drug administration designed for malaria against headlice in Mopeia, Mozambique: a cluster randomised controlled trial
Source: Infect Dis Poverty. 2025 Mar 27;14:25. doi: 10.1186/s40249-025-01290-z (PMC11948683; doi:10.1186/s40249-025-01290-z)
Supplement: Supplementary file 5 — Supplementary Material 5 [file 40249_2025_1290_MOESM5_ESM.docx]

Additional File 5. Indirect effects of iMDA on headlice in children under 5 who were not eligible to take the study drug

|  | **N** | **Headlice prevalence** | **Unadjusted OR** | **p-value** | **Adjusted OR*** | **p-value** |
| --- | --- | --- | --- | --- | --- | --- |
| Control | | | | | | |
| 3 months | 8/120 | 6.67 (2.92-12.71) |  |  | 1 (REF) |  |
| 6 months | 7/122 | 5.74 (2.34-11.46) |  |  | 1 (REF) |  |
| Humans | | | | | | |
| 3 months | 5/109 | 4.59 (1.51-10.38) | 0.72 (0.10-5.10) | 0.743 | 0.75 (0.14-4.00) | 0.740 |
| 6 months | 9/110 | 8.18 (3.81-14.96) | 1.81 (0.44-7.46) | 0.412 | 0.87 (0.16-4.61) | 0.867 |
| Humans and Livestock | | | | | | |
| 3 months | 6/101 | 5.94 (2.21-12.48) | 1.11 (0.17-7.17) | 1.10 (0.16-7.49) | 0.923 | 0.71 (0.14-3.56) |
| 6 months | 7/103 | 6.80 (2.78-13.50) | 1.17 (0.21-6.59) | 1.43 (0.26-8.01) | 0.684 | 0.72 (0.11-4.77) |
